# Hydrophobia Treated by the Poison of the Viper

**Published:** 1844-10

**Authors:** 


*Hydrophobia treated by the poison of the Viper.*—Prince Louis Bonaparte, it appears, has been able to separate the active principle of the poison of the viper, which he has named *echidnine.* He has made the result of his labors known in a memoir, which he read before the chemical section of the congress. In this memoir he proposes echnidnine as a remedy for hydrophobia, and gives an account of an experiment which was made by his request at the hospital of Santa Maria Nuova, at Florence. Six vipers were made to bite a patient laboring under hydrophobia. Death took place, however, with all the symptoms of hydrophobia, without any of those which follow the bite of the viper having occurred. Prince Louis, however, thinks that the active principle of the poison, freed from all other substances, might prove more efficacious if injected into the veins of a person laboring under hydrophobia. The preparation of this substance (echnidnine) is extremely difficult.

The remedy (!) proposed by Prince Louis is not new. It has been tried by Bellingeri at Turin, and the patient died, with the symptoms of the viper bite, well marked, superadded to the hydrophobia which previously existed. Prince Louis states that no such symptoms occurred in his case; but Dr. Griffa maintained that the history of it given by Prince Louis proves the reverse. For our part, we think the trial of such a substance as a remedial agent on man, perfectly unjustifiable, even in so hopeless a disease as hydrophobia. We know not on what ground it could rationally be supposed capable of curing hydrophobia, but we should say, if it must be tried, let its efficacy first be tested on rabid animals, of which, unfortunately, there is no lack.

